# Addressing Health Care Access Disparities Through a Public Health Approach to Physical Therapist Practice

**DOI:** 10.1093/ptj/pzae136

**Published:** 2024-09-17

**Authors:** Jessica McKinney, Nicole Kelm, Brett Windsor, Laura E Keyser

**Affiliations:** Mama LLC, Canton, Massachusetts, USA; Andrews University, Berrien Springs, Michigan, USA; Department of Physical Therapy, Northern Arizona University, Flagstaff, Arizona, USA; Mama LLC, Canton, Massachusetts, USA; Department of Physical Therapy and Rehabilitation Science, University of California, San Francisco, San Francisco, California, USA

**Keywords:** Public Health, Health Services Accessibility, Rehabilitation, Technology

## Abstract

As the field evolves as a doctoring profession, the role and scope of physical therapist practice must also grow to meet important and urgent public health needs. Scalable, population-level interventions must be prioritized to the same degree as tailored, individual-level care. Drawing from public health frameworks, this perspective proposes an approach to population–level physical therapist care delivery that aims to mitigate disease and disability and improve health outcomes by expanding access, decreasing cost, and improving quality of care for those facing the greatest health disparities. Application of these frameworks prompts the development of novel approaches to rehabilitation service delivery to advance twin goals of promoting access to care and reducing health disparities. This paper describes how a population health framework and public health approach can be used to support necessary evolution and innovation within the field of physical therapy and to improve rehabilitation service delivery. Rapid developments in the digital and virtual health space have created a unique opportunity for physical therapists to lean into a new vision of their role as clinicians within the broader health ecosystem. This paper will provide clinicians with a broader perspective of physical therapist expertise and describe opportunities for the development and application of a physical therapist skill set toward driving population health outcomes. Real-world examples will guide clinicians to consider opportunities in their own practice for implementing this public health approach and potentially addressing various contributors to persistent health disparities.

## Disparities in Rehabilitation Access: The Imperative for Change

Globally, the enormous need for rehabilitative services is well-documented, with a recent study in the *Lancet* reporting that 1 in 3 patients worldwide have a condition that would benefit from rehabilitation services.^1^ In years of life lived with disability (YLD), there was a 69% global increase from 1990 to 2019 for key rehabilitation-sensitive conditions. Musculoskeletal disorders (MSDs) are the leading reason, with lower back pain leading among these.^1^ Population growth, aging, increasing burden of non-communicable diseases (NCDs), decreasing mortality, and resultant increased morbidity are other key determinants of rising rehabilitation needs.^1^^,^^2^

Despite this increased demand for rehabilitation services, significant deficits exist in the absolute and proportional supply of human resources for health and rehabilitation. This refers to all health workers involved in meeting rehabilitation needs. Gaps also exist at varying service levels and practice settings (eg, rural vs urban).^2^ Supply–need disparities exist even within high-income countries, and across low- and middle-income countries (LMICs) where rehabilitation resources are scarce.^2^ The lack of an adequate number of rehabilitation professionals contributes to health disparities both within and between countries, where physical therapists are concentrated in high-income, high-resource settings, leaving populations living in low-income, low-resource areas with significant unmet rehabilitation needs. In addition to geographic disparities, inequities in access to and outcomes of rehabilitation services by race and ethnicity, gender, age, and socioeconomic status are also well-documented.^3^

While evidence supports effectiveness of rehabilitation interventions for many NCDs and chronic conditions, global disparities in access to rehabilitation limits the population health impact of these interventions. There have been significant efforts made to codify and expand rehabilitation services globally. The World Health Organization’s (WHO) Rehabilitation 2030 initiative aims to address this global disparity in access to rehabilitation services by advocating for integrated, people-centered rehabilitation systems.^4^ Rehabilitation 2030 emphasizes the importance of rehabilitation as a fundamental component of universal health coverage. Central to this initiative is the World Rehabilitation Alliance, a collaborative platform comprising governments, professional organizations, and civil society groups working together to advance rehabilitation globally.^5^ Additionally, the rehabilitation competency framework, developed by WHO and partners, outlines essential competencies for rehabilitation professionals, ensuring standardized, high-quality care delivery aligned with global health priorities and patient needs.^6^ Through these strategic efforts, Rehabilitation 2030 endeavors to strengthen health systems and improve outcomes for individuals requiring rehabilitation across the lifespan.

Building on these goals, there is opportunity to redefine and reshape the role of care providers, including re-examining the contributions of allied health professionals, such as physical therapists, across the life course and beyond direct clinical interventions delivered in traditional health care settings. While conventional physical therapist practice occurs at the individual level, there is increasing interest and demand for providers to engage with communities and distinct populations to promote and support healthier choices and behaviors in new and more expansive ways.^7^^,^^8^ This is supported by global calls to action for all health care providers to participate in chronic and NCD prevention and management and is most crucial for populations facing the greatest and most disparate burdens of disease.^9^

## A New Way Forward: Physical Therapy and the Public Health Approach

**Figure 1:**
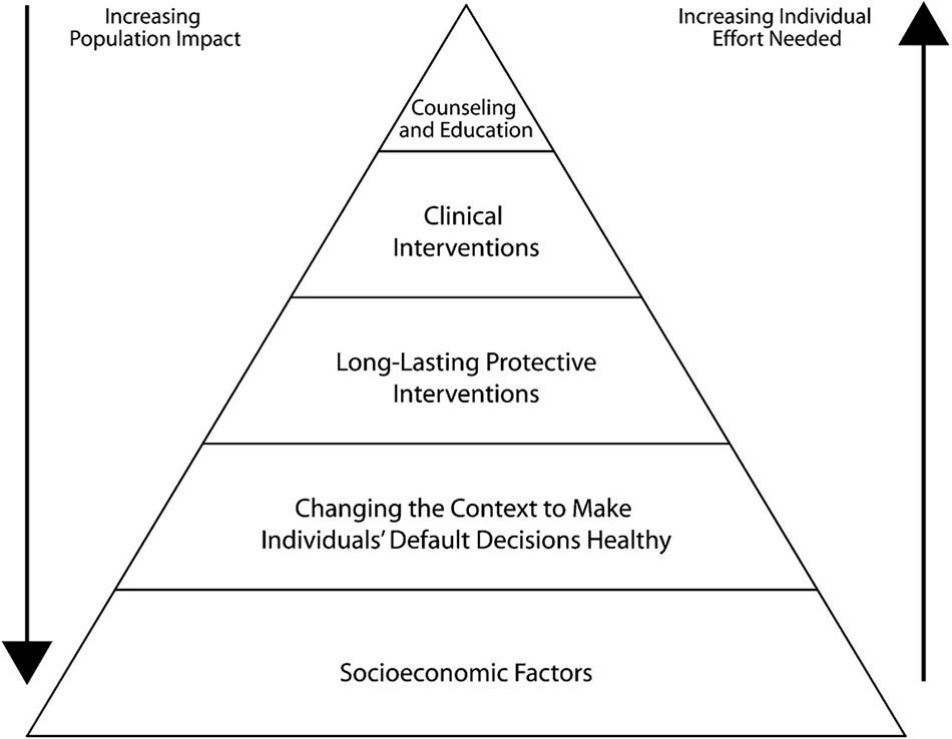
Health impact pyramid. Reprinted with permission from Frieden TR. A framework for public health action: the health impact pyramid. *Am J Public Health*. 2010;100(4):590–595. doi: 10.2105/AJPH.2009.185652.

The health impact pyramid represents a framework for public health action (Fig. 1).^10^ At the base of the pyramid are interventions with the greatest population health impact, and at the top are those with the least. Physical therapist practice has historically operated near the top of this pyramid, focused on tertiary prevention, essentially mitigating the effects of an existing disease, illness, or injury through clinical intervention. Tertiary prevention promotes a continued focus of mastery in physical therapy with priority given to expertise in hands-on skills, health education, therapeutic exercise, and individualized care. Certainly, this level of clinical expertise is necessary and important; yet, such expertise may also be leveraged for the benefit of populations. Further, a broader perspective that includes expertise in primary and secondary prevention and population health management is needed. There is opportunity to apply a physical therapist skill set to the lower levels of this pyramid to affect population health outcomes, reach larger populations, and help to decrease inequities in care and clinical outcomes.^7^^,^^8^

**Figure 2:**
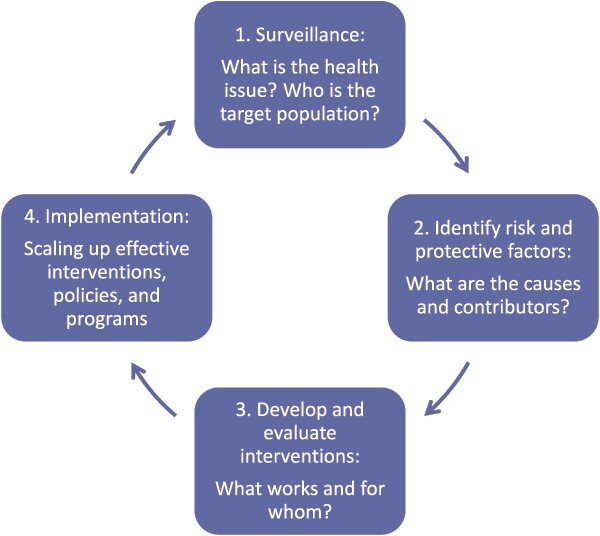
Problem solving in public health: a public health approach to physical therapy.

By embracing health systems thinking, which emphasizes comprehensive and integrated approaches to health care delivery and management, physical therapists can expand their impact beyond individual-level, clinic-based interventions. We support an approach for scaling rehabilitative care to address health care disparities and affect population health by leveraging evidence, expertise, and technology to inform new rehabilitation solutions that are not bound by the constraints of skilled one to one care (Fig. 2). Adapted from problem-solving methodologies in public health, this approach provides a framework for physical therapists to conceive of population-level interventions to expand their reach.^11^^,^^12^ By integrating public health strategies, such as community-based initiatives, health education, and preventive measures, into health systems, physical therapists can enhance their ability to improve population health outcomes.

This integration emphasizes addressing social determinants of health, promoting healthy behaviors, and ensuring equitable access to health care services. Through a synergistic approach that combines systems thinking with public health principles, physical therapists can develop and implement programs that not only treat existing conditions but also prevent the onset of new health concerns. A detailed overview of this approach follows with several examples of real-world applications in the contexts of musculoskeletal health and pelvic floor disorders (PFDs).

1. First, the health problem and population of interest are defined, and relevant epidemiologic information is identified. A population may be defined by a specific geography or region (eg, community, hospital network), by a health condition (eg, obesity, chronic pain), or by a system (eg, employer, military). It is important to define the target population to understand, design, and measure the impact of a particular intervention.
2. Second, causal pathways, risk, and protective factors associated with the health problem are examined. Understanding modifiable and non-modifiable risk factors can be informative in developing interventions that target population subgroups. Research describing potential or known causes of a particular health problem is helpful, particularly for developing health interventions aimed at upstream prevention.
3. Third, evidence-based solutions for the health problem are identified through a review of the literature on health interventions and rehabilitative care. This serves to establish expectations for population health impact that result from a particular intervention and informs selection of key outcome measures for monitoring and evaluation.
4. Implementation is the final step, in which interventions are scaled up, outcomes are evaluated, and new policies or programs are adopted. It is this step that requires the creativity and innovation of the rehabilitation community. New technologies and innovation, including in care models and health promotion, have significant untapped potential to support physical therapists in expanding both the breadth and depth of their interventions.

The circular, iterative nature of this approach allows for ongoing refinement at each step. For example, following implementation, an evaluation may identify a subset of the target population who were not reached by or respond favorably to a specific intervention. Understanding the characteristics of responders and non-responders may be helpful in tailoring the intervention to improve health outcomes. In applying this framework, the non-responders represent another target population with unique risk and protective factors that may require adaptations to the initial intervention or a different intervention altogether. Alternately, there may be a desire to further optimize outcomes among responders or to expand access to different geographies or settings. In each case, this problem-solving approach may be applied to facilitate evidence-based, population-level rehabilitative care.

This framework and expansion of the domain for physical therapy as a discipline may seem daunting, but there are existing applications to leverage and learn from that demonstrate the capacity to improve access and service for marginalized populations and create better outcomes for patients in a variety of contexts. By systematically identifying and addressing the unique needs of different populations, physical therapists can play a critical role in reducing disparities in access to rehabilitative services. This problem-solving approach allows tailoring interventions to the specific barriers faced by underserved groups, whether geographic, economic, cultural, or social. By employing targeted strategies such as community outreach programs, telehealth services, task-shifting/task-sharing, and culturally sensitive care practices, physical therapists can expand access and provide more equitable rehabilitative care. Ongoing evaluation and adaptation of these interventions allow physical therapists to respond more effectively to changing population needs and health landscapes, ensuring sustained improvements in access and health outcomes for all.

## A Unique Opportunity: Digital and Telehealth Innovation

The rise of digital health solutions and expansion of telehealth present significant opportunities to close gaps and advance more equitable access to and quality of rehabilitative care. Telerehabilitation may mitigate costs and increase availability of physical therapist services especially in communities that face access challenges.^13^ Digital health can enable task shifting and provide new mechanisms for remote monitoring and care management to improve adherence and quality of the services provided—thus improving outcomes and reducing disparities. These advantages can also be achieved through more traditional models of task shifting and sharing with other health care professionals coupled with innovations in education and policy.

These shifts have the potential to allow physical therapists to explore innovative financing and expanded holistic care options and partnerships outside of traditional clinical settings, whether through community anchor institutions like schools, employer-based, or in other environments.^14–16^ These tools offer an opportunity to reassess traditional fee-for-service models and explore the value that physical therapists can bring to bear with a much broader interpretation of health care and our role as care providers. Direct access produces significant cost reductions and other positive impacts.^17^ A more expansive view of the role of physical therapists and the tools available to them has the potential to pay similar dividends.

The advent of digital technology in health care has led to a shift in patient preferences, with a growing acceptance of remote care options.^13^ Telehealth has proven advantageous in terms of reduced wait times and increased convenience.^13^ Clinicians are integrating telehealth and in-clinic care, offering a hybrid model that combines convenience and accessibility with comprehensive, personalized care. Data from the Alliance for Physical Therapy Quality and Innovation show that combining telehealth and in-clinic care can extend care plans, leading to improved outcomes and patient satisfaction.^18^ This integrated approach can bridge the gap between rural and urban health care, for example, or provide access for those with disabilities, transportation or other mobility issues, improving access for underserved populations.

Despite the opportunities presented by digital technology, the physical therapy field has been slow to innovate due to regulatory uncertainties.^19^^,^^20^ The COVID-19 pandemic and rapid advancements in digital and virtual health provide an opportunity for physical therapists to redefine their role within the health care ecosystem. Outpatient physical therapist services have traditionally operated within physical locations, but technology and strategic partnerships are enabling channel expansion.^21^^,^^22^ This expansion allows for broader demand, transcending geographical boundaries and time zones, and catering to diverse industries and patient populations.

## Practical Applications: Musculoskeletal Health and Rehabilitation

### Defining the Health Problem and Population of Interest

MSDs are among the most prevalent health issues globally, affecting millions and leading to significant morbidity and disability.^1^ In the United States (US), MSDs affect approximately 1 in 2 adults and are a leading cause of chronic pain, reduced quality of life, and work limitations.^23^ On a global scale, the burden of MSDs is immense, accounting for 1.71 billion cases and contributing substantially to YLD worldwide.^1^ Health disparities in the prevalence and management of MSDs are evident, with LMICs bearing a disproportionate share of the burden.^1^ Factors such as limited access to health care, socioeconomic inequalities, and varying levels of health literacy contribute to these disparities. Additionally, within high-income countries, underserved populations, including racial and ethnic minorities and low-income groups, often experience higher rates of MSD and face significant barriers to receiving effective rehabilitative care.^24^ Addressing these disparities through targeted interventions and equitable health care policies is crucial for improving musculoskeletal health outcomes both in the US and globally.

### Examining Causal Pathways and Risk Factors

MSDs are influenced by a combination of risk and protective factors, which can be biological, environmental, and lifestyle related. Key risk factors include age, with the prevalence of MSD increasing significantly in older adults due to degenerative changes in bones, joints, and muscles.^25^ Sex and gender, as biologic and social determinants of health, also play a role, as those assigned female at birth and women are more likely to develop certain types of MSDs such as osteoporosis and rheumatoid arthritis. Occupational hazards, such as repetitive movements, heavy lifting, and prolonged periods of sitting or standing, contribute substantially to the development of MSD, particularly in jobs requiring manual labor or sedentary office work. Additionally, lifestyle factors like physical inactivity, poor nutrition, obesity, and smoking are strongly associated with an increased risk of MSD. Conversely, protective factors that can mitigate the risk of developing MSD include regular physical activity and adequate nutrition and sleep. Ergonomic interventions in the workplace, such as adjustable workstations and proper lifting techniques, can also prevent MSD.^25^ Furthermore, early intervention and management of symptoms through physical therapy and other rehabilitative services can prevent the progression of MSD and improve overall musculoskeletal health.

There are significant disparities across both risk and protective factors that should be cared for as we identify and design solutions. For example, personal protective equipment for workplace hazards is typically designed for men making this equipment less comfortable and less frequently used by women, increasing the potential for workplace injuries.^26^ Households with a low-income in the US are at higher risk of being food insecure and are at even greater risk for no or low access to healthy and affordable food, poor diets, nutritional deficiencies, and worse health outcomes than households with a higher income.^27^

### Identifying Evidence-Based Solutions

Evidence supports physical therapy as the initial treatment for patients with MSD. However, despite proven clinical outcomes and cost-savings associated with physical therapy, barriers have prevented its widespread adoption and led to disparities between patients based on social identities such as income level or race (eg, utilization disparities between Black and White patients).^28–30^ Additionally, studies have shown that many patients miss physical therapist appointments, with certain social and educational variables predicting higher no-show rates.^31^

The integration of telehealth into the service delivery model enables a full continuum of musculoskeletal services with a physical therapist first approach. Patients who receive physical therapy as their first choice tend to be more satisfied, leading to reduced cancelations and no-shows. These patients also respond more quickly and achieve better treatment outcomes, reducing the need for expensive medical interventions.^17^^,^^28^ The hybrid model of telehealth and in-clinic care not only improves clinical outcomes but also has the potential to broaden the scope of services to include nutrition, sleep, stress management, and spirituality, promoting holistic care.^32^ By integrating a public health framework into physical therapy, the field can address issues of accessibility, quality of care, and health costs, reducing disparities and promoting health equity. Outpatient physical therapy is well-positioned to connect in-clinic, onsite, and virtual solutions for MSD, offering a comprehensive approach. Partnerships with digital health providers enable immediate access to injury education and care programs, with licensed physical therapists available online for consultations and triaging to appropriate care providers. Additional digital health technologies include sensors, wearables, and health apps, which may be leveraged for MSD management.^33^^,^^34^ Outside of digital health, task-shifting and task-sharing approaches can also improve access to care for individuals with MSD. This may include optimizing the role of physical therapist assistants or physical therapist aides, training community health workers on key aspects of rehabilitative care or working within a health care system to streamline direct access to physical therapy, including nesting services within primary health care centers or providing emergency room consults.^22^^,^^35^^,^^36^

### Implementation and Evaluation

Increasingly, employers in the US recognize the benefits of contracting directly for health services, which allows them to create health care programs tailored to their workforce’s specific needs, potentially leading to improved employee health outcomes, reduced health care costs, and higher employee satisfaction.^14^^,^^15^ Outside the US, some LMICs are using elements of the direct contracting model to improve utilization of health services and outcomes.^16^ This trend toward direct contracting is particularly relevant in rural areas, where the challenges of health care access and cost are often more acute. For example, rural school districts, with their small employee numbers and limited budgets, are especially vulnerable to sudden, high-cost health claims and ongoing expenses associated with MSD. A case study from a small school district in rural Wisconsin illustrates the potential benefits of this approach.^37^ Over 2 years, this district contracted directly with a large outpatient physical therapist provider. The school district promoted the program to all employees and facilitated easy access to physical therapist services, including telehealth options and a digital-led hybrid care model. This initiative led to substantial cost savings: a 30% decrease in opioid prescriptions, a 21% reduction in non-physical therapist MSD expenses, a 35% reduction in outpatient hospitalizations, and a 50% reduction in emergency room visits and inpatient surgeries.^37^ These benefits were achieved without compromising care quality or utilization rates and were accompanied by significant increases in patient and employee satisfaction.

Another example from the Netherlands highlights the effectiveness of a systems approach to NCD management that emphasizes the role of specialized physical therapist services, while reducing barriers to rehabilitation access. Chronic CareNet is a Dutch nationwide network of specialized physical therapists established to provide high-quality supervised exercise therapy and lifestyle counseling for patients with NCDs.^38^ Physical therapists working within Chronic CareNet must meet quality criteria, including baseline training and continuing education, and the resulting specialization covers multiple NCDs. The network leverages technology infrastructure that includes an online care finder and referral system. Personal therapist accounts and a quality monitoring system ensure continuous improvement and transparency. Regional networks foster collaboration and knowledge exchange among therapists. The network ensures that patients receive the right care in the right place, tailored to their specific needs and conditions, which helps to address disparities. Early data suggest that this model increases patient adherence to exercise therapy and lifestyle changes. The online care finder and referral system ensures patients are quickly connected to specialized therapists, reducing delays in treatment initiation. Data shows high rates of adherence to treatment guidelines and improved patient outcomes, such as increased walking distance and quality of life. The model also shows potential cost savings for NCDs by reducing health care utilization through effective management and prevention strategies.^38^

Given the significant prevalence and disabling nature of MSD, these represent just some examples of innovation within rehabilitative care for the affected population by applying health systems’ thinking and a public health approach to improving health outcomes and reducing inequities. Coupling digital and telehealth tools with innovations in provider education, service platforms, reimbursement, and contracting models allows for an expansion of service availability at lower costs in ways that reduce barriers around health literacy, proximity, transportation, and social needs for those facing the greatest inequities.

## Practical Applications: Women’s Health and Pelvic Floor Disorders

### Defining the Health Problem and Population of Interest

PFDs include urinary incontinence, fecal incontinence, and pelvic organ prolapse and are estimated to affect at least a third of women worldwide.^39^^,^^40^ PFDs disproportionately affect women and those with female reproductive organs, are sufficiently prevalent to warrant consideration as a population health concern, and have multilevel gaps in care.^39^^,^^40^ Urinary incontinence is the most prevalent and researched PFD, with both prevalence and severity increasing with age.^41^ There are approximately 28 million women in the US and hundreds of millions worldwide with bothersome urinary incontinence symptoms.^40^^,^^41^ Urinary incontinence conveys negative economic, physical, and psychosocial impacts and is associated with decreased quality of life and physical activity, anxiety and depression, and at older ages, with functional decline and sarcopenia.^42–47^

### Causal Pathways, Risk, and Protective Factors

Pregnancy and childbirth are the most significant risk factors for PFDs, including urinary incontinence, contributing to their recognition as maternal morbidities.^39^^,^^48^ Disparities in maternal health care and birth outcomes increase risk. This is particularly evident in low-resource settings, where lack of access to maternal health care, including safe surgery, contributes to obstetric fistula, a severe maternal morbidity resulting in continuous urinary incontinence and/or fecal incontinence.^49^ Additional risk factors for urinary incontinence are obesity, chronic cough, chronic constipation, aging, and smoking.^39^^,^^50^^,^^51^

Though health disparities data related to PFDs is limited, research suggests these conditions are associated with experiencing greater social needs (eg, food insecurity, financial strain, un- or underemployment, housing insecurity) as are higher out-of-pocket incontinence management costs.^52–56^ Pooled prevalence of health service utilization is low at 37%, with Asian and Black women and those for whom symptoms were normalized or for whom fear or misinformation were factors serving as barriers to care.^57^ Brown and Simon call for applying a health equity lens to urinary incontinence highlighting structural vulnerability due to language proficiency, for example, for Indigenous and Black women, and for rural populations.^58^

### Identifying Evidence-Based Solutions

Evidence exists for a stepwise approach to urinary incontinence treatment that begins with conservative management.^59^ The first step in this care pathway most clearly applies to rehabilitation. Pelvic floor muscle training (PFMT), a program of “exercises for improving pelvic floor muscle strength, endurance, power and/or relaxation”, is supported by Level 1 evidence as the core of first line care for most urinary incontinence, along with health education.^60^ The best outcomes have been reported when PFMT is supervised (eg, 1 time per week and taught and monitored by a health professional, clinician, or instructor).^61^ Group-based PFMT and unsupervised PFMT have been found feasible and effective for urinary incontinence.^62^^,^^63^ Professional societies have endorsed urinary incontinence screening and initiation of care, including PFMT, via telehealth, and evidence has been published in favor of telerehabilitation for urinary incontinence treatment.^64^^,^^65^ A scoping review of digital health technologies focused on mobile apps found Level 2 evidence in support of their utility in urinary incontinence treatment.^66^

Most women, in high- and low-resource settings, alike, will never participate in treatment for their urinary incontinence and, like those with other PFDs, will live with the negative and often progressive nature of their condition for their entire lives.^67^ Individual, institutional, and structural barriers contribute to this gross undertreatment.^68–71^ Studies describing utilization of care found that of women referred to physical therapist services for PFDs, including urinary incontinence, approximately 50%–60% initiate care by attending a single visit.^72^^,^^73^ Of those who initiate care, there is further fall off, whereby approximately 30% complete 3 or more visits. A retrospective claims analysis found that in the 2 years following an initial diagnosis of urinary incontinence, less than 3% had documented PFMT or physical therapist visits associated with their diagnosis.^74^ These data highlight major and problematic implementation gaps in the rehabilitation services known to be effective in treating urinary incontinence. When a public health framework is applied to this case, several solutions ready for implementation at scale emerge.

### Implementation and Evaluation

It is in the fourth phase of applying a public health approach that focuses on implementation and scaling up where the most work is needed. Physical therapists are involved in telerehabilitation, group-based care, and product development, including in the emerging areas of FemTech and digital health. With the rise of wearable technologies, remote monitoring and app-based tracking and education, physical therapists can digitize and scale interventions, expanding rehabilitation access without compromise to quality of care or programmatic rigor. Digital solutions to scale up PFMT present opportunities to serve patients in new ways. One evidence-based, physical therapist–informed digital therapeutic solution is the *Leva* Pelvic Health System (Axena Health, Inc; Auburndale, MA, US), a combination of hardware and software that leverages motion-based feedback mechanism and app-based health education, adherence monitoring, and symptom tracking. This digital health product has demonstrated superior clinical results in comparison to an at-home PFMT program at an 8-week primary end point, demonstrated durability of results at 24-months, regardless of ongoing exercise, and has published real-world evidence of effectiveness.^75–77^

New hybrid service and educational approaches can help to address access and cost barriers that create inequities in resource constrained or lower income settings with learnings for those in other environments. In a recent women’s health rehabilitation project that centered care for women with fistula along with other PFD in sub-Saharan Africa, a variety of health workers were engaged in rehabilitation task-shifting and task-sharing, employing both group and individual level care. Subsequent work developed an open-access training guide for health workers that focused on pelvic health and rehabilitation; it is hosted online in 4 languages and has been downloaded in over 25 countries.^78^ The training guide serves as a standalone resource to enhance the capacity of individual clinicians and facilities provision of rehabilitative care, including health education and structured physical therapist training. Additionally, a training-of-trainers was conducted in Nigeria in response to updated national guidelines on fistula care, which explicitly name the need for physical therapists to participate in holistic care and calls for their involvement in training other health workers in rehabilitative skills to increase capacity for treating women with fistula, in line with WHO recommendations.^6^^,^^79^

First line care for urinary incontinence is rehabilitative care and should be led by the rehabilitation community. However, this can take a variety of forms and leverage all available resources, including digital and technology-enabled solutions, new service delivery platforms, and service extenders such as other health workers. The digital, educational, and service extension solutions described here are well-positioned to scale horizontally and improve access to care, especially those of lower socioeconomic status, disadvantaged racial or ethnic groups, or facing geographic barriers, while addressing the critical shortage of skilled specialized providers.

## Conclusion: An Expansive and More Equitable Future

There is significant opportunity to be found in applying health systems and public health approaches to the physical therapist field. We have outlined how the health impact pyramid can help physical therapists to apply their skills at lower levels of the pyramid with a view toward prevention and providing access to interventions at scale, while improving the circumstances in which individuals make choices about their health and access to care. We have also described a problem-solving approach that may be useful in developing and expanding physical therapist interventions. Application of these frameworks present opportunities for growth of the discipline, innovation in the field and ultimately, improvements in access, quality, and cost for patients with the greatest needs.

The use cases presented here represent real-world and evidence-based implementation strategies that serve to expand and scale access to care. There are the obvious benefits of trends such as digital health that can expand access to and shift the context of physical therapist services. As we improve our ability to provide population level care, our value as a doctoring profession increases with physical therapists presenting as the provider of choice through both new and existing platforms. If we can develop population health solutions, it will improve our ability to meet health needs in a challenging economic environment and develop new revenue streams and offerings, while reaching larger volumes of patients including those that have historically faced disparities due to gender, race and ethnicity, geography, disability status, age, and other characteristics or identities. We encourage physical therapists to consider leveraging these tools and frameworks in service of the populations under their care. Relying on highly individualized and in-person care through traditional clinical settings alone cannot meet the needs of populations and will continue to leave too many people behind.
